# Body Mass Index and Frailty Among Older Mexican Americans: Findings From an 18-year of Follow-up

**DOI:** 10.1093/geroni/igab046.2993

**Published:** 2021-12-17

**Authors:** Megan Rutherford, Brian Downer, Chih-Ying Li, Soham Al Snih

**Affiliations:** 1 The University of Texas Medical Branch, Galveston, Texas, United States; 2 University of Texas Medical Branch, Galveston, Texas, United States

## Abstract

The objective of this study was to examine body mass index (BMI) as predictor of frailty among non-frail Mexican American older adults at baseline. Data are from an 18-year prospective cohort of 1,647 non-institutionalized Mexican American aged ≥ 67 years from the Hispanic Established Population for the Epidemiologic Study of the Elderly (1995/1996-2012/13). BMI (Kg/m2) was grouped according to the National Institutes of Health obesity standards (<18.5=underweight, 18.5–24.9=normal weight, 25.0–29.9=overweight, 30.0–34.9=obesity category I and ≥ 35=obesity category II and extreme obesity). Frailty was defined as meeting three or more of the following: unintentional weight loss of >10 pounds, weakness, self-reported exhaustion, low physical activity, and slow walking speed. Covariates included socio-demographics, comorbidities, cognitive function, depressive symptoms, and limitations in activities of daily living (ADL). General Estimating Equations were performed to estimate the odds ratio (OR) and 95% confidence interval (CI) of frailty as a function of BMI categories. All variables were analyzed as time varying except for gender and education. Participants in the underweight or obesity type II / morbidity obesity category had increased OR of frailty over time than those in the normal weight category (2.68, 95% CI=1.46-4.9 vs.1.55, 95% CI=1.02-2.35, respectively) after controlling for all covariates. Those who reported arthritis, hip fracture, depressive symptoms, or ADL disability had increased odds of frailty over time. This study showed a U-shaped relationship between BMI and frailty over an 18-year period of follow-up which has implications for maintaining a healthy weight to prevent frailty in this population.

